# Majority and popularity effects on norm formation in adolescence

**DOI:** 10.1038/s41598-021-92482-8

**Published:** 2021-06-18

**Authors:** Ana da Silva Pinho, Lucas Molleman, Barbara R. Braams, Wouter van den Bos

**Affiliations:** 1 Department of Psychology, University of Amsterdam, Amsterdam, The Netherlands; 2 Amsterdam Brain and Cognition, University of Amsterdam, Amsterdam, The Netherlands; 3 Faculty of Behavioral and Movement Sciences, Department of Clinical, Neuro- and Developmental Psychology, Vrije Universiteit Amsterdam, Amsterdam, The Netherlands

**Keywords:** Psychology, Evolution

## Abstract

Personal norms consist of individuals’ attitudes about the appropriateness of behaviour. These norms guide adolescents’ behaviour in countless domains that are fundamental for their social functioning and well-being. Peers are known to have a marked influence on adolescent risk-taking and prosocial behaviour, but little is known about how peers shape personal norms underlying those behaviours. Here we show that adolescents’ personal norms are decisively moulded by the norms of the majority and popular peers in their social network. Our experiment indicates that observing peer norms substantially impacts adolescents’ normative evaluation of risk-taking and prosocial behaviours. The majority norm had a stronger impact than the norm of a single popular peer, and norm adjustments were largest when adolescents observed strong disapproval of risk-taking or strong approval of prosocial behaviour. Our study suggests that learning about peer norms likely promotes adolescents to hold views and values supporting socially desirable behaviour.

## Introduction

Humans are uniquely successful in coordinating behaviour, fostering economic exchange, and maintaining fruitful social relationships in large, highly complex societies. A key factor underlying these features is people’s strong reliance on social norms—typical patterns and rules that govern individual behaviour^1–3^. People often follow social norms even in the absence of sanctions, or when it is not in their immediate self-interest to do so. Even though norms vary considerably across societies^4^ and generations^5^, their importance seems universal^6^. Understanding how norms arise is particularly relevant in the context of current social and global challenges, such as rising inequality, climate change, and the rapid development of digital technologies^7^. Such challenges may constitute an opportunity for norm dynamic changes specifically during the time individuals explore new environments and prepare for an independent adult role in society^8,9^.

Adolescence is widely viewed as a crucial period for the emergence of norms^10^ and shifts in existing ones^11,12^. During this formative developmental stage, individuals’ decisions become grounded in personal norms, that is, their attitudes about the appropriateness of behaviour in given situations^13,14^. Personal norms reflect individuals’ perceptions of social expectations that may be enforced through punishments and rewards^15^. At the same time, peers become prominent sources of influence on adolescent development and decision-making^16,17^. Little is known, however, about how peers shape incipient personal norms during adolescence. In this study, we show that adolescents’ personal norms are shaped by four factors: (i) the social source (popular peers or majority of peers), (ii) the adolescent’s age, (iii) the domain of behaviour (risk-taking and prosociality), and (iv) the direction of the peer norm (more moderate or more extreme peer disapproval or approval relative to one’s initial personal norm).

As children transition into adolescence, belonging to the peer group becomes increasingly important^18^, and learning about peer norms becomes essential to navigate the various new social contexts they encounter^19^. Individuals can infer relevant norms by observing prevalent behaviour in their social network^20^, or can learn them more directly when peers communicate their approval or disapproval^21^. Observational studies of peer influence on behaviour point to two key sources of influence within social networks: popular peers and the majority^22–25^. In this paper, we examine the idea that these social sources may also impact adolescents’ personal norms. Conforming to peer norms can help adolescents fit in with their peer group by choosing group-appropriate behaviour^26^. Indeed, seminal work on social influence has shown that group conformity represents an effective way of lessening social sanctions and conflict, and maximizing gains within a social group^27^. In addition, popular peers are highly visible and well-connected in the peer group, and their personal norms may accurately reflect group norms^11^. Following the majority, however, may be even more informative and effective given that it consists of multiple peers^8^, reflecting the social norm. That is, the social norm consists of aggregated personal norms within the peer group. We therefore expect popular peers to be influential, but that majority norms have a larger impact on personal norms. In addition, based on previous empirical studies we expect the impact of popular peers to be particularly strong around early- or mid-adolescence and to decline with age^23,28–30^.

Peer influence in adolescence has traditionally been associated with undesirable effects, such as increased risk-taking and antisocial conduct^9,31,32^, but recent studies show that peers can also promote prosocial behaviour^25,30,33^. Studies in this area typically focus on the effects of peers on adolescents’ behaviour rather than the personal norms underlying these behaviours^34–36^. However, they do allow for deriving hypotheses about how peers might drive adolescent norm formation in the domains of risk-taking and prosocial behaviour. First, we expect that adolescents’ personal norms generally indicate disapproval of risk-taking and approval of prosocial behaviour. However, how peer norms impact adolescents’ personal norms in these domains likely depends on their direction. That is, peer norms may show more moderate or more extreme disapproval or approval than one’s personal norm. Recent evidence suggests that risk-averse peers impact adolescent behaviour more strongly than risk-seeking peers^36,37^. We accordingly hypothesise that extreme peer disapproval of risky behaviour more strongly impacts adolescents’ personal norms than moderate peer disapproval. The scant behavioural evidence in the domain of prosocial behaviour does not suggest such an asymmetry^34^, so we do not have specific expectations about the relative impact of extreme versus moderate peer approval of prosocial behaviour on adolescents’ personal norms.

**Figure 1:**
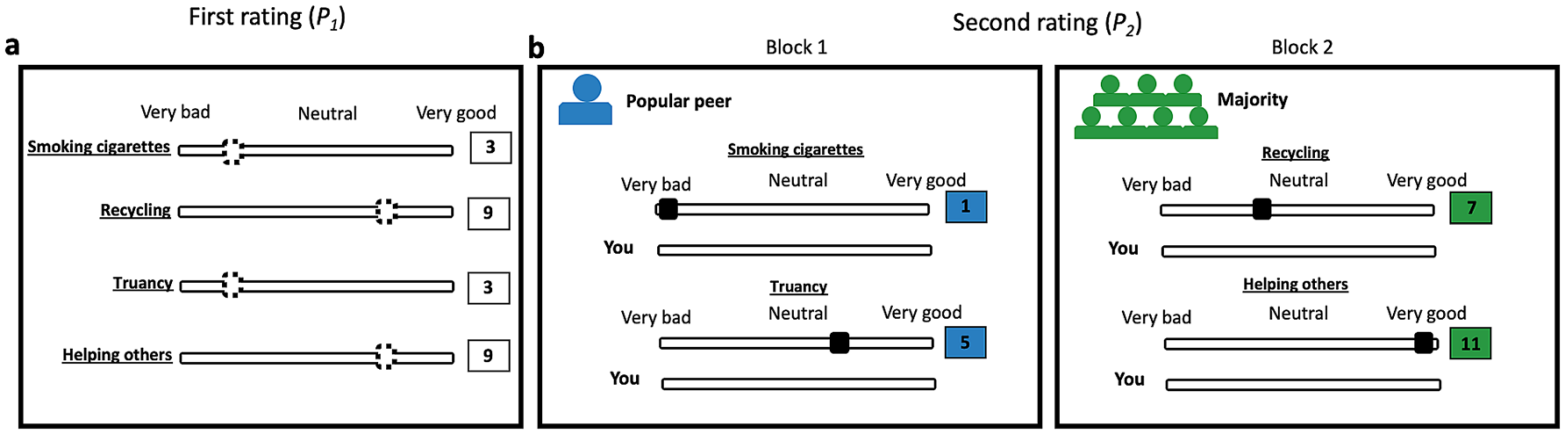
Experimental design. (**a**) In wave 1, we measured participants’ personal norms (*P*_*1*_) as their rating of a set of risky and prosocial behaviours (dotted handle on the sliders; ranging from 1 = very bad to 11 = very good). (**b**) In wave 2, participants observed normative information (*NI*) and rated their personal norms again (*P*_*2*_). Our key variable of interest is the peer impact on personal norms, calculated as *S* = (*P*_*2*_ − *P*_*1*_*)/(NI* − *P*_*1*_*)*. The task included two experimental blocks representing either a popular peer or the majority, and which order of appearance was counterbalanced. A trial represented a given behavioural item to rate. Each block contained 6 trials, 3 reflecting risk-taking behaviours, and 3 reflecting prosocial behaviours. Within each block, we varied the direction of normative information: 2 trials in which the information was more moderate than *P*_*1*_, 2 trials in which the information was more extreme than *P*_*1*_, and 2 filler trials in which the information was the same as *P*_*1*_ (see “Methods” for definition of wave 2 trials).

Our pre-registered study addresses a set of key outstanding questions, using a controlled two-wave experimental design (Fig. 1). In the first wave, we measured adolescents’ personal norms of a set of risk-taking and prosocial behaviours (see Table S1 for a full list). Additionally, we constructed their social network in five high school classrooms (*N* = 89 adolescents, ages 12 to 19) to identify the social sources. In the second wave, adolescents observed the personal norm of a popular peer or the norm of the majority of their peers and rated their personal norms again. Specifically, the normative cues were equally available from each social source and balanced in terms of their direction in both domains (i.e., more extreme or more moderate disapproval or approval than one’s initial rating), while holding constant the distance between initial ratings and the normative information (see Methods for details). The adjustment in ratings between wave 1 and wave 2 quantified the impact of peers on personal norms. We examined how this impact depended on the adolescents’ age, the direction of the peer norm and the domain of behaviour. Our experimental setup allowed us to test the influence of two social sources, popular peers and the majority on adolescents’ personal norms, using real norms within their social networks, while avoiding the challenges of identifying peer effects in observational studies (e.g., selection and reflection problems^38,39^).

Our results reveal that adolescents’ personal norms are strongly influenced by normative information derived from peers in their social networks. The impact of the majority was very high among early adolescents but declined with age. The impact of popular peers was somewhat weaker but remained stable across ages. Importantly, norm adjustments were largest when adolescents were exposed to extreme peer disapproval of risk-taking or extreme peer approval of prosociality. Our results suggest that learning about peer norms may promote desirable outcomes as it increases adolescents’ disapproval of risk-taking and increases their approval of prosocial behaviour.

## Results

### Adolescents’ personal norms prior to peer influence

As expected, participants’ personal norms in wave 1 vastly differed by behavioural domain. Risk-taking behaviours were generally disapproved of (individuals’ mean rating across all risk-taking items: 3.13, s.d. = 0.97 on an 11-point scale; range 1–5) and prosocial behaviours were generally approved of (mean: 8.45, s.d. = 0.98; range 6–11; Fig. S1a). Older adolescents were less disapproving of risk-taking behaviours than younger adolescents (*F* (2, 86) = 24.88, MSE = 0.61, *p* < 0.001; Fig. S2), and we observed no statistically significant age trend with respect to prosocial behaviour (*F* (2, 86) = 0.61, MSE = 0.96, *p* = 0.547). Taken together, these results indicate that, on average, risk-taking behaviours were disapproved of and prosocial ones approved of.

### Majority norms trump the norms of popular peers

**Figure 2:**
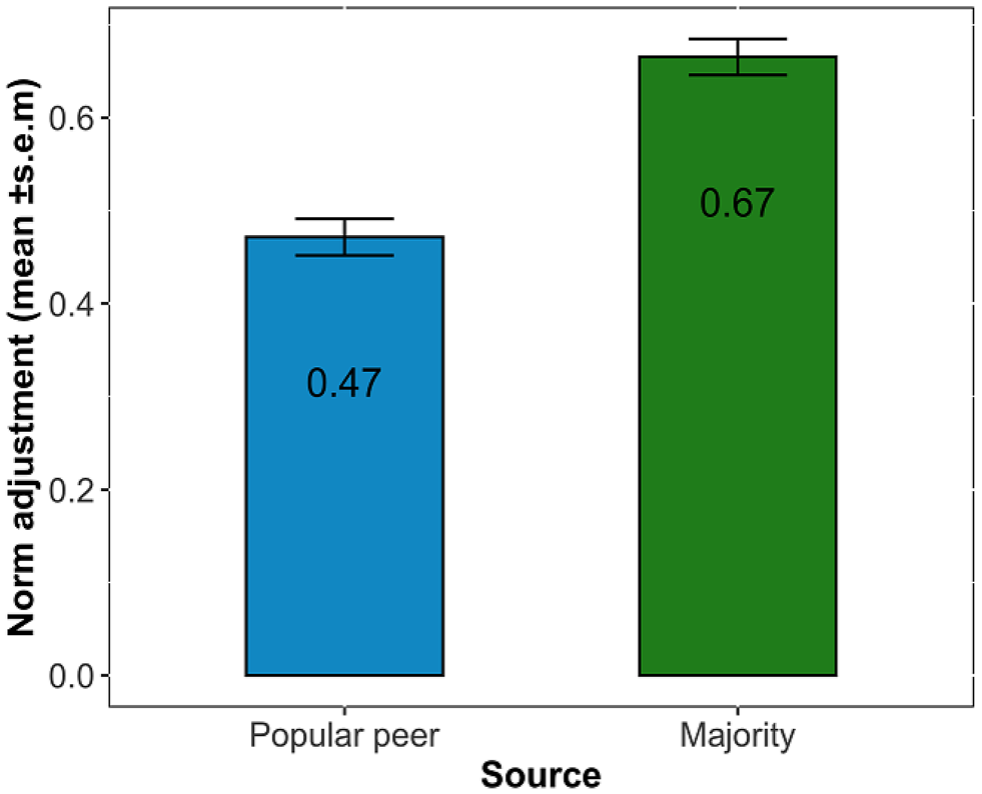
Peer norms impact adolescents’ personal norms. Bars show participants’ norm adjustments from wave 1 to wave 2 that were averaged across trials, broken down by social source. Error bars indicate ± s.e.m*.*

We examined the effect of the social source (i.e., popular peers and the majority) on participants’ norm adjustments and whether this effect varied across ages. Overall, normative information strongly impacted participants’ personal norms (Fig. 2). When normative information was provided by a popular peer, participants adjusted by 47% on average, indicating that they assigned almost equal weight to the norm of a popular peer and their own initial norm. When normative information was provided by the majority, participants adjusted even more, assigning twice as much weight to the majority norm than to their own (67% on average; linear mixed-effect model, main effect of ‘source’, regression *β* = 0.19, 95% CI = (0.13, 0.24), Table 1, model 1). The impact of social source on adjustments depended on age (interaction effect, regression *β* = − 0.07, 95% CI = (− 0.12, − 0.01), Table 1, model 1). Specifically, the effect of the majority decreased with age, while the impact of popular peers remained stable across ages (Fig. S3). Overall, these results indicate that relative to popular peers, the majority has a strong impact on adolescents’ personal norms.

**Table 1:** Determinants of participants’ norm adjustment.

	Dependent variable:	
	Norm adjustments	
	Model 1	Model 2
(Intercept)	0.47 (0.43, 0.51)	0.55 (0.49, 0.61)
	*p* = 0.00	*p* = 0.00
Source (1 when Majority, 0 when Popular peer)	0.19 (0.13, 0.24)	0.16 (0.11, 0.21)
	*p* = 0.00	*p* = 0.00
Age (continuous)	− 0.01 (− 0.04, 0.03)	− 0.01 (− 0.05, 0.03)
	*p* = 0.79	*p* = 0.63
Source X Age	− 0.07 (− 0.12, − 0.01)	− 0.06 (− 0.12, − 0.01)
	*p* = 0.02	*p* = 0.03
Direction: Extreme disapproval risk		0.02 (− 0.05, 0.09)
		*p* = 0.64
Direction: Moderate disapproval risk		− 0.16 (− 0.23, − 0.08)
		*p* = 0.0001
Direction: Moderate approval prosocial		− 0.13 (− 0.21, − 0.06)
		*p* = 0.001
Number of participants	89	89
Observations	692	692

Linear mixed-effects models were fitted to norm adjustments, using ‘participant’ as random intercept. Model 1 tested whether participants’ norm adjustments were influenced by the social source (popular peer or majority) and whether this effect was dependent on age (hypothesis 1). Model 2 tested whether participants’ norm adjustments were influenced by the direction of the peer norm (more moderate or more extreme peer disapproval or approval relative to one’s initial personal norm) and the domain of behaviour (hypothesis 2). Both models report unstandardized coefficients. The 95% confidence intervals (CI) are in parentheses and *P* values below the CI.

### Extreme norms trump moderate ones

**Figure 3:**
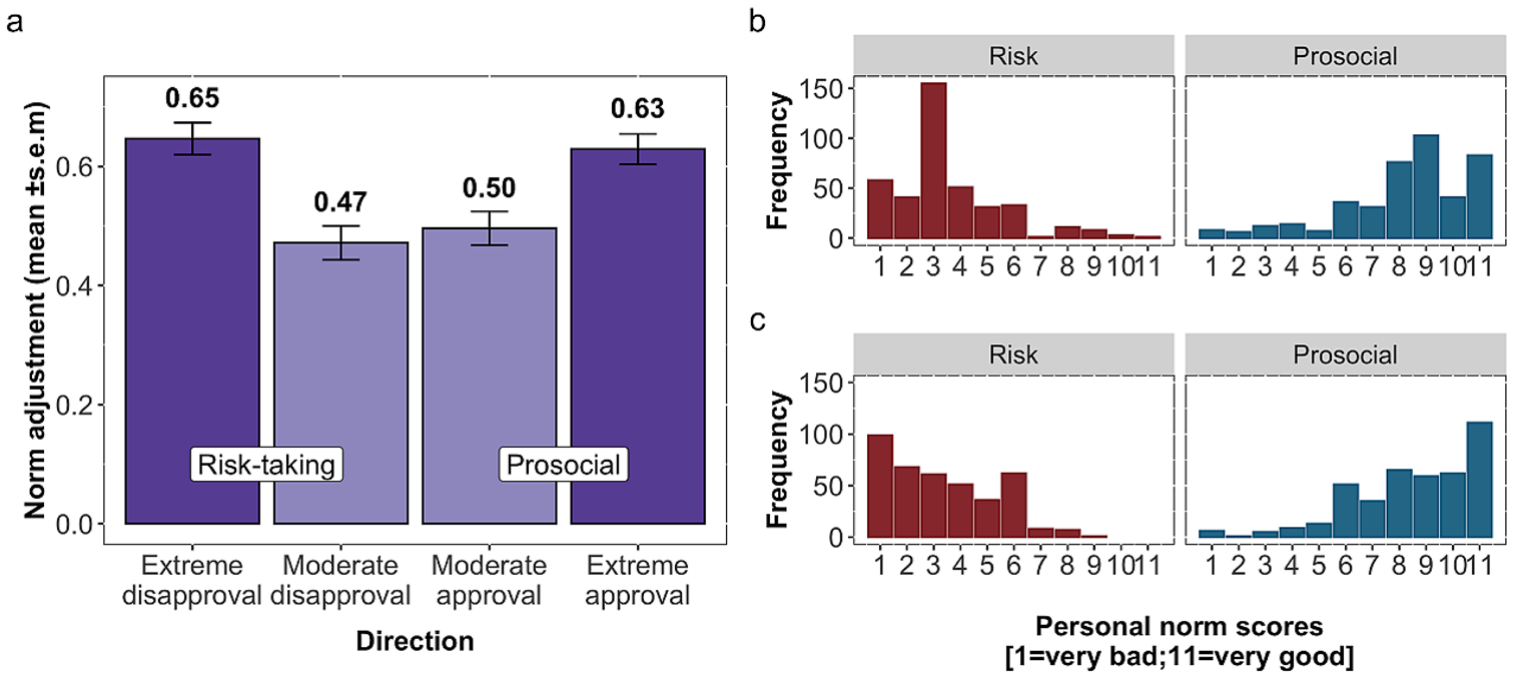
Impact of peer norms depends on their direction and the domain of behaviour. (**a**) Bars show participants’ mean adjustments, broken down by behavioural domain (risky versus prosocial behaviour) and direction of peer norms (more moderate and more extreme peer disapproval or approval relative to participants’ initial personal norm). Error bars indicate ± s.e.m*.* (**b**) Frequency distributions of risk-taking personal norms (red) and prosocial personal norm ratings (blue) from wave 1 (before observing peer norms). Most risk-taking items were rated as moderate disapproval of, while most prosocial items were rated as moderate approval of. (**c**) Frequency distributions of personal norms ratings in wave 2 (after observing peer norms). Participants tended to move their personal norms towards the extremes in both behavioural domains, particularly in risk-taking.

We examined whether participants’ norm adjustments were influenced by the direction of the observed norm (i.e., more moderate or more extreme peer disapproval or approval relative to participants’ initial personal norm) and whether this effect differs between behavioural domains (i.e., risk-taking and prosociality). The impact of the direction of the peer norm on adjustments depended on the domain of behaviour (Fig. 3a). In the risk-taking domain, participants adjusted their norm by 65% on average when the normative information expressed extreme disapproval than their initial personal norms, and by 47% when it expressed moderate disapproval. In prosocial behaviour, participants adjusted their norm by 63% on average when it expressed extreme approval relative to participants’ baseline scores, and by 50% when it expressed moderate approval. The impact of the direction of the peer norm was corroborated by a linear mixed-effect model showing that moderate peer norms had considerably weaker effects on adjustments than extreme ones did (reference: extreme approval prosocial; moderate disapproval risk regression *β* = − 0.16, 95% CI = (− 0.23, − 0.08), and moderate approval prosocial regression *β* = − 0.13, 95% CI = (− 0.21, − 0.06), Table 1, model 2). That is, peer norms had the greatest impact when they signalled strong disapproval of risk-taking or strong approval of prosocial behaviour.

The relatively strong impact of extreme peer norms resulted in an overall shift of adolescents’ personal norms into socially desirable directions (Fig. 3b,c). Prior to observing peer norms, adolescents tended to disapprove of risk-taking behaviour (Fig. 3b, red bars), but this disapproval grew stronger after observing peer norms (Fig. 3c, red bars). Conversely, adolescents initially tended to approve of prosocial behaviours (Fig. 3b, blue bars), but this approval grew stronger after observing peer norms (Fig. 3c, blue bars). The overall changes of adolescents’ personal norms occurred under a balanced presentation of normative content (i.e., extreme vs moderate disapproval of risk-taking or approval of prosocial cues) while holding the distance between their initial norms and normative information constant. These results suggest that relative to moderate peer norms, extreme peer norms substantially shape adolescents’ personal norms of behaviour.

### Norm updating strategies

**Figure 4:**
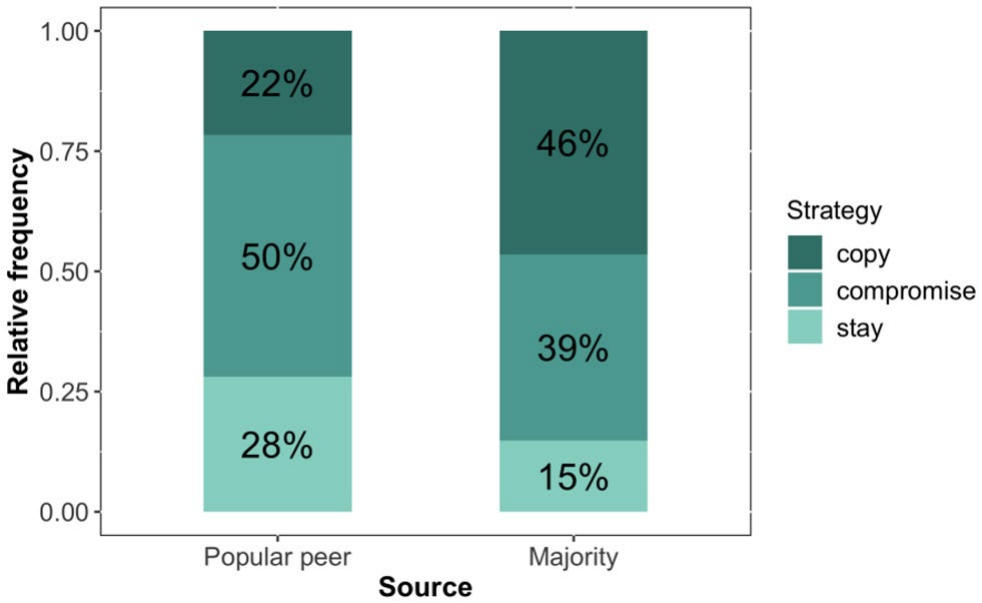
Participants’ norm updating strategies. Stacked bars show the distribution of norm updating strategies used by participants when exposed to normative information. The way they applied these strategies differed considerably when observing information provided by a popular peer and the majority from their social network.

To obtain a more detailed understanding of the processes underlying adolescents’ norm adjustments, we explored the strategies they used to integrate normative information to form their personal norms. Most norm adjustments in individual trials could be grouped into three strategies^40,41^: *stay* (keeping the initial rating, *S* = 0), *copy* (adopting the normative information provided, *S* = 1), and *compromise* (moving the rating in-between stay and copy, 0 < *S* < 1). When normative information was provided by a popular peer, participants kept their rating in 28% of the trials, compromised in 50% and copied the source’s rating in 22% (Fig. 4). When normative information was provided by the majority, participants kept their rating in 15% of the trials, compromised in 39% and copied the majority rating in 46%. Logistic generalized mixed models revealed that the type of social source did not impact the likelihood of staying, but participants were more likely to copy the norm expressed by the majority (Table S2, model 1 and 2). Furthermore, older participants were more likely than younger participants to keep their initial personal norm when information was provided by the majority, which is consistent with their lower average norm adjustments (Table S2, model 1). With respect to the *copy* strategy, we observed no statistically significant age trends for either social source. Additionally, we observed an effect of the direction of the peer norm and domain on *copy* strategy (Table S2, model 2). Participants were less likely to copy moderate peer norms than extreme peer norms of risky and prosocial behaviours. Overall, these results indicate that participants are more likely to copy the majority norm and extreme peer norms in both behavioural domains, which is in line with their substantial norm adjustments.

## Discussion

Our study examined how adolescents’ personal norms are shaped by norms in their social network. We present four main findings. First, the norms of both popular peers and the majority of peers in the classroom impacted adolescents’ personal norms. Second, adolescents’ personal norms were influenced more strongly by the majority than by popular peers. Third, the effect of popular peers’ norms remained stable across ages, while the impact of the majority norm decreased with age. Fourth, norm adjustments were largest when adolescents observed strong disapproval of risk-taking or strong approval of prosocial behaviour. Together, these findings show that learning about peer norms has the potential to promote the formation of socially desirable norms during adolescence.

Adolescents’ norm adjustments (Fig. 2) were considerably larger than those reported in the adult literature^41–44^. Adults typically tend to put more weight on their own views than that of others^42^. In our study, adolescents assigned at least equal weight to the norms of their peers than to their own. This finding is consistent with previous studies that have suggested an increased sensitivity to peer influence during adolescence^45^. The substantial norm adjustments may be explained by the fact that we provided cues from a real and relevant peer group, whereas most previous studies, including those with adults, used anonymous others as sources. Information from adolescents’ peer groups is more relevant for them compared to socially distant sources. Another possible reason for favouring normative information over one’s beliefs could be the lack of exposure to adolescents’ own initial ratings while being confronted with peer norms. This feature of our task may have contributed to more salient normative information compared to other experimental designs that include prior beliefs. Yet, this lack of the explicit previous rating may at the same time be closer to real social interactions that involve receiving someone else’s opinion.

Our experimental approach enabled us to compare the effects of popular peers and the majority. The majority norm had a greater effect than the norm of a single popular peer (Fig. 2). At first glance, these results appear to contradict previous research suggesting that high-status peers may be more impactful than the majority on adolescents’ norms and attitudes^22^. However, this discrepancy can be explained by the differences in availability of the norms between studies. While information about the norm of a single popular peer may be learned more easily (given that they are highly visible in the social network and are likely to voice their views), normative information from the majority may be more difficult to estimate given that it requires gathering and integrating more pieces of information. Our experiment controlled for the availability of the normative information, thus allowing us to compare the magnitude of their impact, while avoiding potential confounds imminent in observational approaches (e.g., distance effects, reflection and selection issues^39^). One crucial feature of our experimental design is its comparative nature. In future studies it would be interesting to include a wider variety of social sources, such as unpopular peers, direct friends, or distant friends. Here we focused on the two sources that are theoretically seen as very salient in adolescence, but such extensions would provide further insight in the relative impact of different social sources.

In line with our expectations, the effect of the majority norm decreased with age (Fig. S3; Table 1, model 1). However, contrary to our expectations, the effect of popular peers remained stable with age (Fig. S3). This finding has two important practical implications for norm dynamics within adolescents’ social networks and the school context. First, it underlines the importance of interventions based on revealing true group norms, if these reinforce desirable norms or weaken negative ones. Second, it highlights the importance that popular peers can play in interventions as it appears that their norms seem to be highly influential^46,47^, and that their influence may stay strong even in late adolescence. The latter can be particularly cost-efficient given that it would only require changing the norm of a single individual, rather than of a whole group of individuals to achieve intended outcomes.

We also observed that adolescents’ personal norms were more strongly impacted by extreme peer norms than moderate norms (Fig. 3). That is, norm adjustments were largest when adolescents observed extreme peer disapproval of risk-taking or extreme peer approval of prosocial behaviour. This result contradicts the popular notion that peer influence predominantly causes a negative impact on adolescents’ development^31^. Instead, our findings support the idea that adolescents are more willing to align their own norms in a socially desirable manner (i.e., higher levels of disapproval of risk-taking and more approval of prosocial behaviours) when provided with information from their peers. This finding is in line with recent findings in the experimental literature on social learning in the context of risky decisions^35,36^. Despite this positive pattern, it is possible that perceived social norms still have an overall negative impact due to biased perception. That is, adolescents tend to systematically overestimate how their peers think about certain behaviours, in particular risk-taking (e.g., they think that their peers approve more and/or smoke and drink more than they actually do^48–50^). In this context, when popular individuals are overly risk-seeing^51^, majority norms can be highly beneficial. In turn, prosocial norms can be set by popular peers in case they express high levels of prosociality.

While our experimental task allowed us to investigate the effect of peer norms on adolescents’ norm formation in a highly controlled and rigorous manner, our findings may not entirely translate the complexity and ambiguity of social norms in real-life settings. For instance, extreme norms may be more easily communicated given that they tend to be more explicit than moderate norms. The latter norms may take more time and be more difficult to convey and interpret, relying on other features of norm communication (e.g., facial expressions, gestures, voice intonation). Although it was beyond the scope of our study, future research could investigate adolescents’ conformity to social-conventional and moral information derived from their social networks and examine whether they follow the same conformity patterns as those previously found with children^52^. Additionally, although the identity of the demonstrators conveyed the norm was anonymized, adolescents may have formed an opinion about the popular peers in their class. Future empirical work could address the role of individuals’ own preference/ liking of popular peers and test whether it mediates the impact of peer norms on adolescents’ personal norms. Lastly, future research could investigate whether adolescents’ personal norms change when they are exposed to peer norms that are opposite to their own (e.g., approval vs. disapproval).

Overall, our findings indicate that communication of peer norms within adolescents’ real social networks can influence their personal norms in positive ways. Observing peer norms lead adolescents to increase their disapproval of risk-taking and increase their approval of prosocial behaviour. Given that personal norms impact adolescents’ behaviour^14^, changing personal norms may contribute to desirable behavioural changes (i.e., less risk-taking and more prosocial behaviour). The current study provides promising evidence that may help tailor preventive strategies for peer norm misperceptions and inform interventions that aim to weaken risk-taking norms and reinforce prosocial norms.

## Methods

### Participants

We recruited 176 participants from eight classrooms from a Dutch high-school. Participants from classrooms in which participation rates were lower than 50% were excluded from the main analyses. The reason for this exclusion criterion was that we could not robustly identify the majority or popular peers to determine the conditions of our experiment. Hence, three classes were excluded from the main analyses, resulting in a sample of 112 participants. The exclusion of these classrooms could have resulted in a selection bias. For instance, one may believe this led to the exclusion of groups of individuals who are less group-oriented, resulting in biased findings, by selecting classes more susceptible to group conformity. However, the decisions to participate or not were made independently, and participation rates were often dependent on the lack of parental consent. The sample was further reduced due to failure to complete the task within the given time, drop-outs or absence in the wave 2 of our study. As a result, the final sample consisted of 89 participants (66% identified as female, mean age = 14.8, sd = 2.1) from five classrooms. In terms of high-school years these classes were divided into two classes of 1st year of high-school, one of 3rd and two of 5th. Although we have a smaller group of participants in the 3rd year of high-school, we did not exclude these data from the age-related analyses given that we expected linear age effects.

To understand whether the excluded classes followed the same pattern as the included data, we checked participants’ personal norms in wave 1. Comparable to the final sample, participants from the excluded classes generally disapproved of risk-taking behaviours (individuals’ mean rating across all risk-taking items: 2.68, s.d. = 1.27 on an 11-point scale; range 1–5) and approved of prosocial behaviours (mean: 8.05, s.d. = 1.18; range 6–11; Fig. S1b).

### General procedure

Prior to the study, we obtained informed consent from all participants. For minors, we additionally obtained informed consent from their parents or legal guardians. The study took place at a Dutch high school in a classroom setting. Data were collected in 2 waves with a three-week interval. The classroom setup was the same as for a regular class, except for the study equipment on the students’ study tables. Before each testing session, the experimenters placed tablets and screen dividers on the tables so students had enough privacy to perform the experiment. Each session started with instructions, followed by possible questions about the study. After that, participants completed a battery of questionnaires and tasks on the tablets. Each session lasted approximately 45 min and schools received a monetary compensation of €5 per participant for each wave. Additionally, participants could obtain points in some tasks to incentivize performance. Participants’ points were converted into lottery tickets and one participant per class could win a €40 online shop voucher. The experiment was developed with the software LIONESS^53^. All procedures were approved by the Ethics Review Board Faculty of Social and Behavioral Sciences of the University of Amsterdam (case number 2019-DP-11305) and performed in accordance with relevant guidelines and regulations.

### Experiment—wave 1

Stimuli consisted of a set of 36 items displaying risky and prosocial behaviours (see Table S1 for a full list). Participants had to rate their approval of each of those behaviours using a slider ranging from 1 (very bad) to 11 (very good). The order of the items was randomized between participants. Participants’ ratings in wave 1 were used to define the normative information in wave 2 and to compute the measure capturing the adjustments in participants’ personal norms (for pre-registered study setup, hypotheses and predictions, see https://osf.io/cx2u6).

### Experiment—wave 2

Participants had to rate 12 out of 36 items from wave 1 again; 6 reflecting risk-taking and 6 reflecting prosocial behaviours. The screen for entering ratings also displayed normative information (but not their own wave 1 rating). We believe that personal norms are held on one’s values and should be easily accessible without individuals being reminded of their prior beliefs. That is, we tried to create a more naturalistic setting in which normative information of others is communicated to us but we are not often explicitly confronted simultaneously with our opinions. Nevertheless, not proving participants’ initial ratings may have caused more pronounced peer norms compared to other designs that include prior beliefs.

In each experimental block consisting of either a popular peer or the majority (presented in a counterbalanced order), we varied the direction of the normative information (2 trials indicating more extreme, and 2 trials indicating more moderate (dis)approval than the participant’s rating in wave 1; see Supplementary Methods for screenshots of the task). As a control, we also included 2 filler trials per block in which peer norms were the same as the participant’s wave 1 rating. On average, participants’ absolute change between the two waves was 0.97 in filler trials and 2.82 in normative information trials, indicating that participants moved way more in the task conditions.

Majority information reflected the mode rating for an item, and was communicated to participants as the “*most chosen rating in your classroom*”. Ratings of popular peers were obtained by selecting the rating of a peer who was among the top-five of most nominated classmates on the question “which of your classmates is the most popular?”. This was communicated as “*the rating of a popular classmate”*.

To determine wave 2 trials, we targeted participants’ initial disapproval of risk-taking (*P*_*1*_≈3) and approval of prosocial behaviour (*P*_*1*_≈9), holding constant the distance between *P*_*1*_ and *NI* (distance≈2 ratings higher or lower than their first rating). Peer disapproval of risk-taking behaviour was either more extreme (*NI*≈1) or more moderate (*NI*≈5) than participants’ initial personal norms. Peer approval of prosocial behaviour was more moderate (*NI*≈7) or more extreme (*NI*≈11) than participants’ initial personal norms (see Fig. S4 for details on how we programmed the stimuli in wave 2). Overall, risk-taking and prosocial behaviours were balanced across experimental conditions. Out of 692 observations included in the main analysis, 336 reflected risk-taking behaviours (157 moderate and 179 extreme) and 356 reflected prosocial behaviours (160 moderate and 196 extreme). Our experiment did not use deception; the normative information provided to participants was real and from their social networks. However, given the sensitivity of some behavioural items we did not show the actual name(s) of the demonstrator(s) but instead the social sources as described above.

For both waves, the on-screen instructions included illustrations, practice sliders, and compulsory comprehension questions to ensure that participants understood their task (see Supplementary Methods for screenshots). Although the gist of the task instructions was identical between waves, instructions regarding the assessment of the behavioural items were slightly more extensive in wave 2. We did this to make participants’ task in this wave more straightforward given that they would be exposed to normative information from popular peers and the majority. We underline that this may have not impacted the difference in the social conditions (popular peers vs majority) but may have made some responses more extreme given the emphasis on the appropriateness of behaviour (whether it is good or bad). Nonetheless, low average moves in filler trials do not suggest more extreme responses between waves.

### Assessing participants’ sensitivity to normative information

For each trial in the task, we computed peer impact on personal norms: *S* = (*P*_*2*_ − *P*_*1*_*)/(NI* − *P*_*1*_*),* where we computed the difference between participants’ ratings in wave 2 and wave 1 and divided it by the difference of normative information observed and participants’ ratings in wave 1. The value of *S* can be viewed as the weight participants assign to normative information relative to participants’ initial ratings in forming their second rating: *P*_*2*_ = *(1* − S)*P_*1*_ + S * NI. A value of *S* = 0 indicates ignoring normative information and keeping one’s initial personal norms, *S* = 0.5 indicates assigning equal weight to one’s initial norms and normative information, and *S* = 1 indicates copying the normative information observed. In line with expectations, in an overwhelming majority of cases, participants’ second rating was a weighted average of their first rating and normative information (0 ≤ *S* ≤ 1), and our main analyses focused on these cases only^54,55^ (692 individual rounds). Cases of anticonformity (*S* < 0; 7% of cases, representing 54 out of 793 rounds) and moving beyond the normative information (*S* > 1; 5% of cases, representing 47 out of 793 rounds) were rare. These cases correspond to individual rounds from different participants and represent qualitatively different types of behaviour in a particular round, that is the second rating is not a weighted average of the individual’s existing personal norm and normative information. Therefore, these individual rounds were removed from the analyses.

### Statistical analyses

All statistical analyses were conducted in R statistical software, Rstudio v. 1.3.1093. We used linear mixed-effects models for all main analyses. These statistical analyses were performed using the *lmer* function of the *lme4* package^56^. Linear mixed models used maximum likelihood fitting method and included ‘participant’ as random intercept to account for individual differences in adjustments. The reported effects were based on two-tailed tests.

In our first model we tested whether participants’ norm adjustment was influenced by the source of normative information (popular peers or the majority of peers), and whether this effect was dependent on age (hypothesis 1; Table 1, model 1). In our second model, we tested whether the effect of direction on participants’ norm adjustment was dependent on the domain of behaviour (hypothesis 2; Table 1, model 2).

### Exploratory analyses

We further explored adolescents’ norms by considering three qualitatively different updating strategies^40,41,57^: *stay* with one’s rating, *copy* the normative information or *compromise* by adjusting one’s initial rating towards normative information. We first examined participants’ frequency of adjustments in individual trials according to these strategies (Fig. S5). Then, we investigated participants’ tendency to stay with their initial norm or copy the normative information depending on (a) the source and age, and (b) the direction and domain. The results on strategy use reported in the main text are based on logistic generalized linear mixed models using the *glmer* function of the *lme4* package^56^, separately for participants’ stay and copy strategies.

## Supplementary Information

Supplementary Information.

## Data Availability

All data and code supporting the findings of this study are available from the public repository, accessible via https://github.com/AnaSPinho/norm_formation_in_adolescence.
