# Supplementary material for: Majority and popularity effects on norm formation in adolescence
Source: Sci Rep. 2021 Jun 18;11:12884. doi: 10.1038/s41598-021-92482-8 (PMC8213745; doi:10.1038/s41598-021-92482-8)
Supplement: Supplementary file 1 — Supplementary Information. [file 41598_2021_92482_MOESM1_ESM.docx]

**Supplementary Information for:**

**Majority and popularity effects on norm formation in adolescence**

by

Ana da Silva Pinho, Lucas Molleman, Barbara R. Braams, Wouter van den Bos

#

#

# **Contents**

1. Supplementary Figures
2. Supplementary Tables
3. Supplementary Methods

#

# **Supplementary Figures**


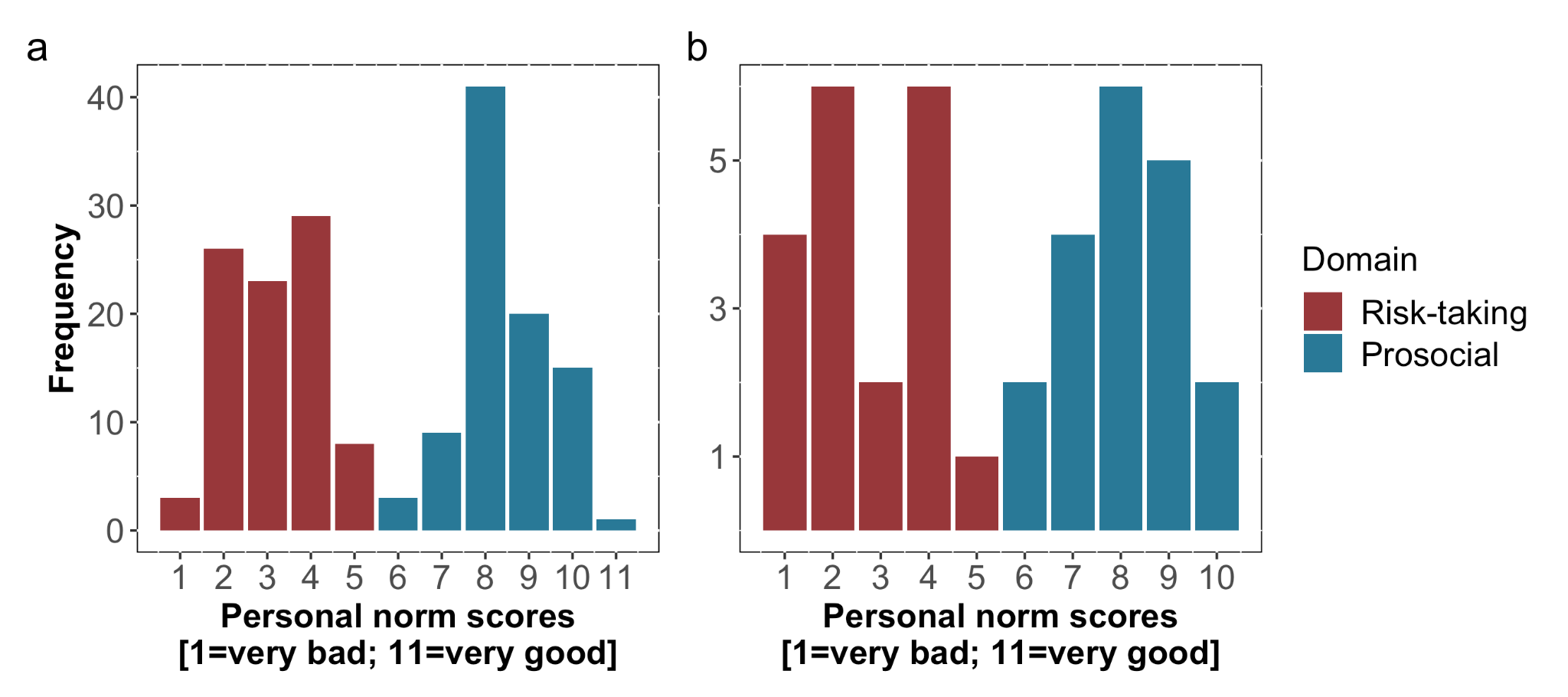


**Fig. S1 | Frequency distribution of participants’ mean personal norms in wave 1.** **a,** Data from participants included in the final sample, where the X-axis shows their mean personal norm scores (1= very bad; 11= very good) of risk-taking (red bars) and prosocial behaviour (blue bars). Low values indicate participants’ disapproval of behaviour and high values approval of it. **b,** Data from participants from the excluded classes show a similar pattern to those in the final sample. Generally, risk-taking behaviours were disapproved of and prosocial behaviours approved of.


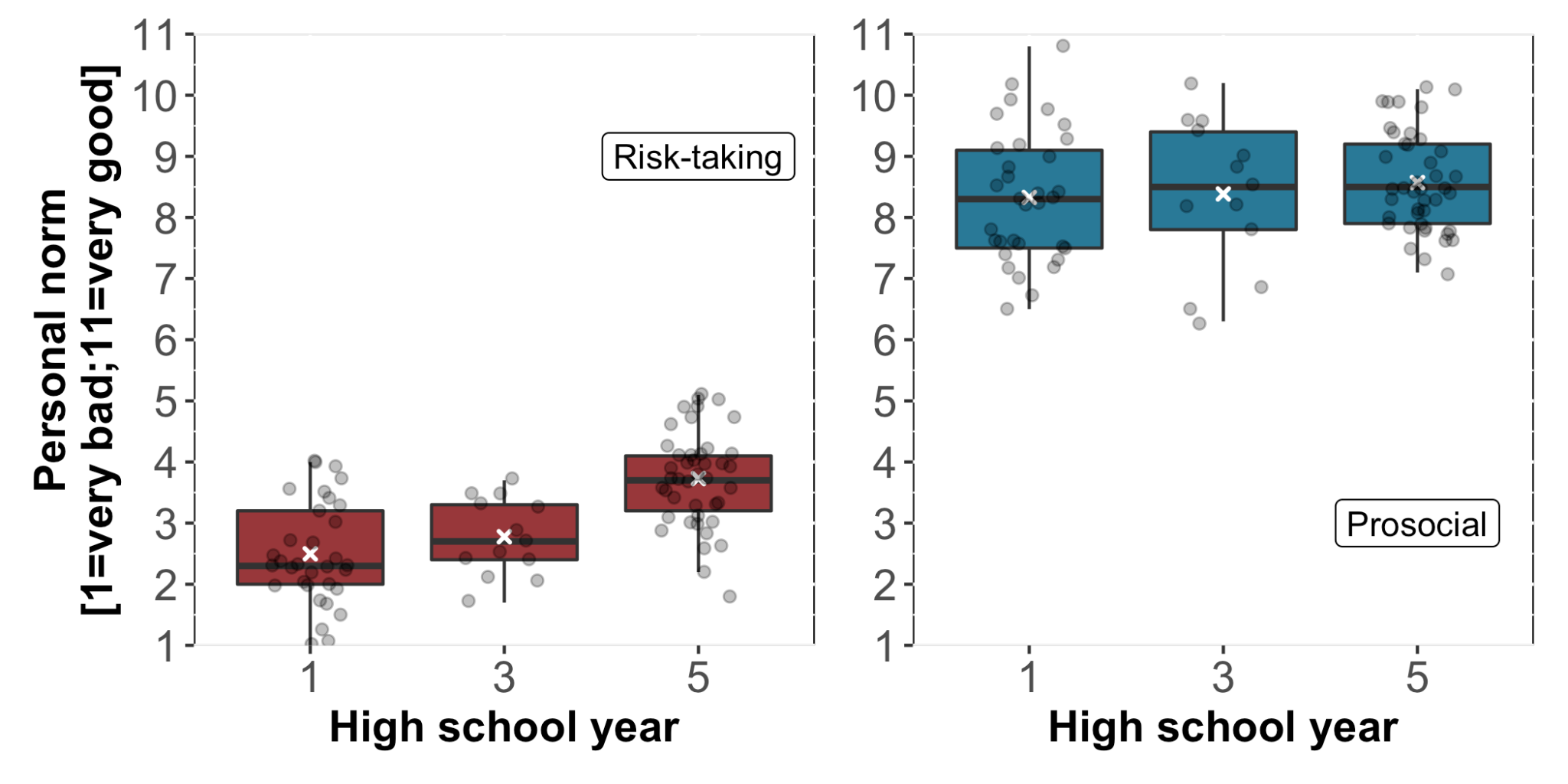


**Fig. S2 | Participants’ personal norms in wave 1 broken down by high-school years.** Participants' mean personal norms of risk-taking (white crosses in the red boxes) increase over high school years, while their mean personal norms of prosocial behaviours (white crosses in the blue boxes) remain relatively stable across these high school years. Grey dots indicate individual participants’ data points. High school first year mean age = 12.8; third year: mean age = 15.4 and fifth year mean age= 16.8.


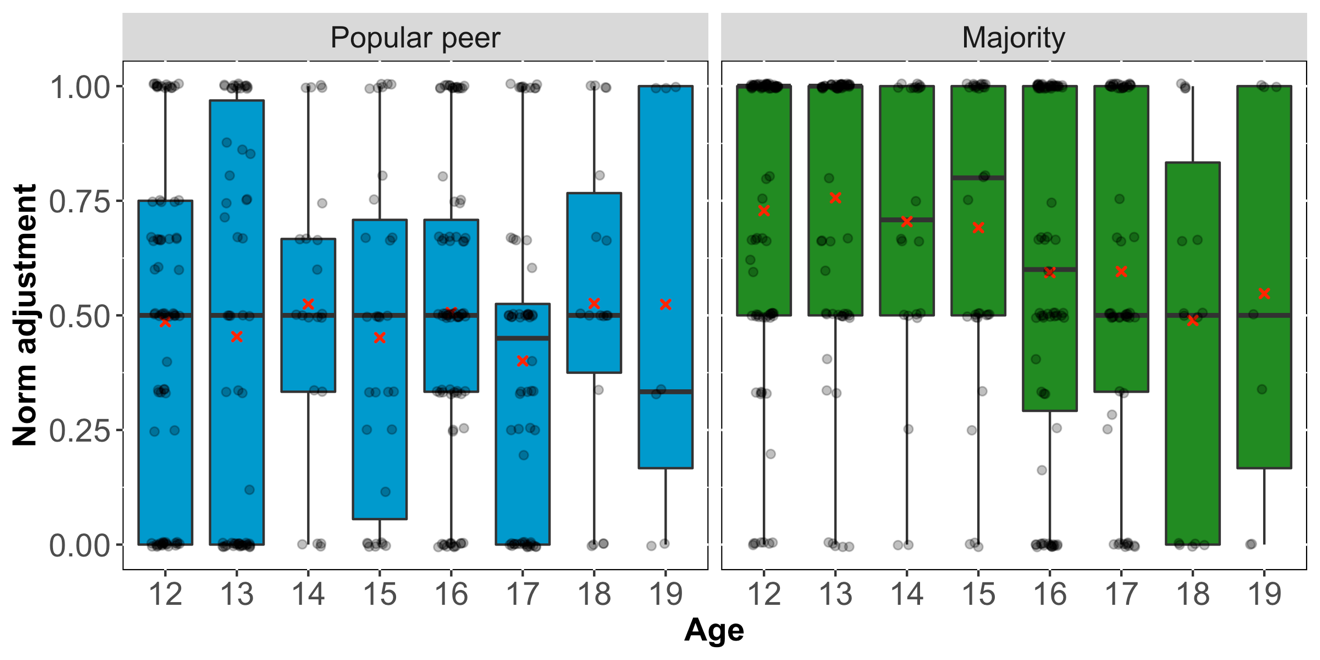


**Fig. S3 | The effect of source of normative information across ages.** The impact of social sources on adolescents’ norm adjustments depends on age. Red crosses indicate mean norm adjustment across ages, and grey dots indicate individual participants’ data points. The effect of the norm of popular peers remains stable during adolescence, while the effect of the majority norm decreases with age.


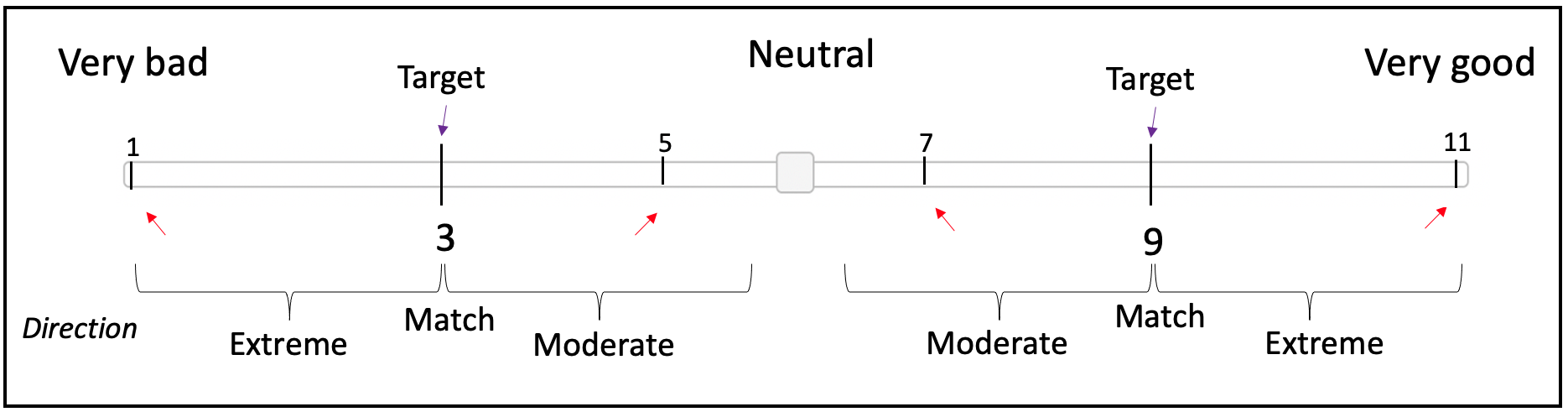


**Fig. S4 | Programming the stimuli for wave 2.** The scheme depicts the process of building up the trials for the stimuli in wave 2 for each experimental block. The task included two experimental blocks representing either a popular peer or the majority. A trial represents a given behavioural item to rate. We selected a subset of behaviours (12 out 36) consisting of 6 risk-taking and 6 prosocial behaviours per participant. The blocks were presented in a counterbalanced order and contained 6 trials each (3 risky and 3 prosocial behaviours; represented by the vertical lines). Within blocks, we varied the direction of the normative information: 2 trials indicating information more extreme and 2 trials indicating more moderate information than participants’ initial personal norms). We targeted participants’ initial disapproval of risky behaviour (≈3) and approval of prosocial behaviour (≈9), holding constant the distance between their personal norms and normative information (distance≈2 ratings higher or lower than their initial disapproval and approval). To determine this distance or as close as possible to 2 ratings apart, we have applied a cost function to deviations from the respective targets (3 and 9). For each target trial (6 trials in which we targeted participants’ disapproval≈3 and 6 trails in which we targeted participants’ disapproval≈9), we selected the normative information derived from each social source (i.e., popular peer vs majority) with the lowest penalty. For instance, to define a *disapproval extreme information trial* in the popular peer block, we would target a participant’s personal norm of 3 and would search for a popular peer (out of the top-five of most nominated peers) who had a given disapproval trial of 1. For each trial and experimental conditions, the process would iterate multiple times until finding the optimal peer norm. If no peer norm would correspond to the given conditions, a penalty would be applied and the next best option would be selected (e.g., target disapproval≈4, extreme disapproval≈2). Most of the trials targeting initial personal norms of 3(9) were successfully selected, but we also retained trials in which low penalties were applied. The remaining 2 trials were used as control and consisted of filler trials in which the normative information was the same as participants’ initial ratings.


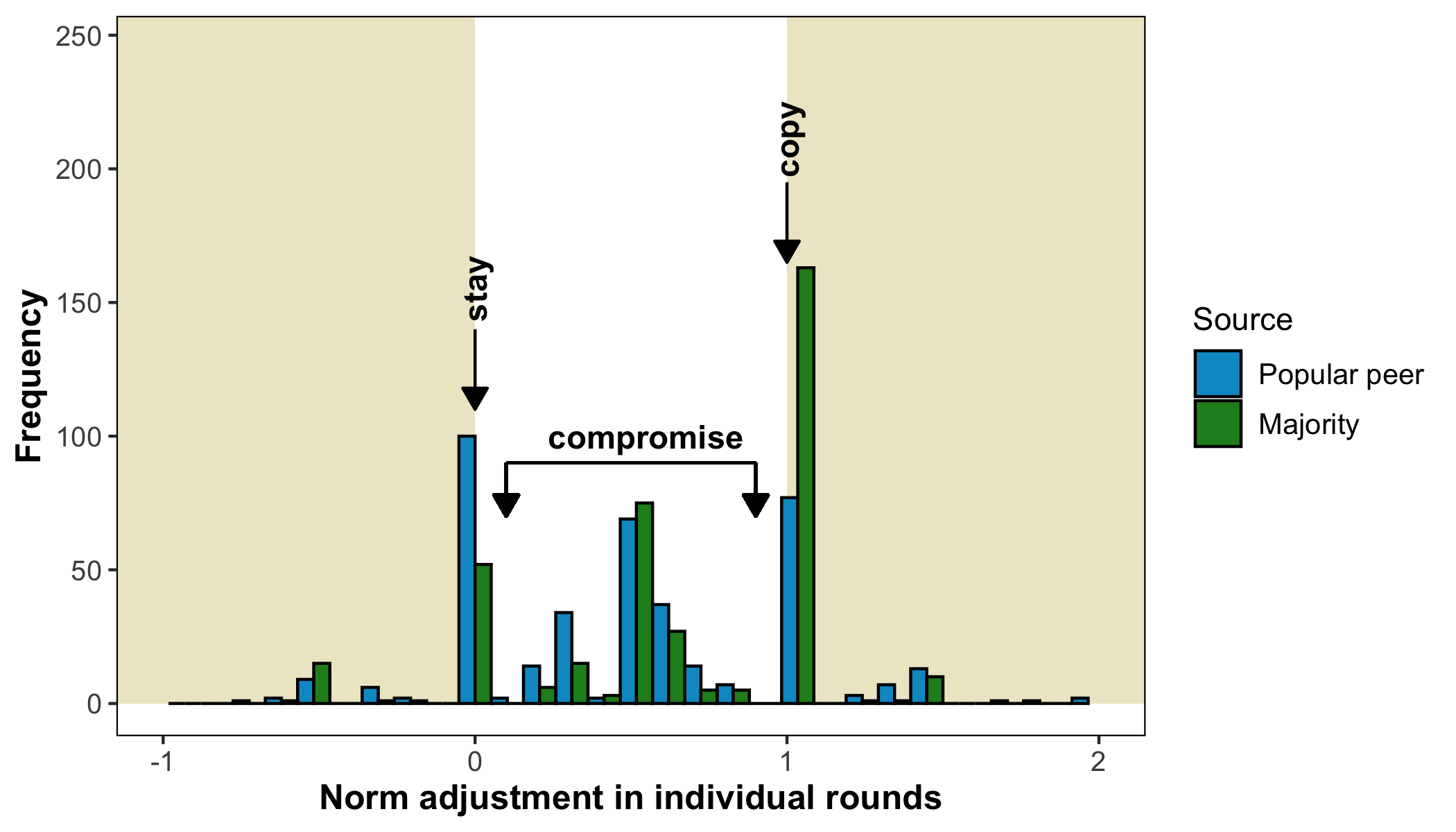


**Fig. S5 | Frequency distribution of norm adjustments in individual rounds**. Participants’ norm adjustments in individual rounds broken down by source of normative information. The X-axis shows the relative adjustment towards normative information computed for each round as: *S*= (*P_2_-P_1_)/(NI- P_1_)*, separately for the norm of popular peers (blue bars) and the norm of the majority (green bar). A value of *S*=0 indicates ignoring normative information and keeping one’s initial personal norms, *S*=0.5 indicates assigning equal weight to one’s initial norms and normative information, and *S*=1 indicates copying the normative information observed. Values of (*S*<0) and (*S*>1) in the tails of the distribution (shadowed) were rare.

# **Supplementary Tables**

## Table S1 | List of behavioural items by domain

| Behavioural domain | | |
| --- | --- | --- |
| Offline risk-taking | Online risk-taking | Prosocial |
| Smoking cigarettes | Downloading illegal software or films | Helping others with problems |
| Drinking alcohol | Playing online games | Standing up for a peer who is bullied |
| Getting drunk | Watching pornographic content online | Being vegetarian |
| Smoking weed | Connecting with strangers on online platforms (e.g., Facebook, Instagram) | Striking for climate |
| Truancy | Using parents credit card without permission to buy things online | Recycling |
| Bullying | Sharing nudes | Donating to charities |
| Cheating on an exam | Watching violent content online | Doing sports |
| Engaging in unprotected sex | Using social media to humiliate a peer | Giving money to homeless |
| Stealing small items from a store | Cyberbullying | Volunteering work (e.g., scouting) |
| Texting or calling while cycling | Spending more than 4hours on social media platforms | Sharing things (food, class notes) |
| Snowboarding without protection | Spending all your allowance on online games | Inviting a peer who is not popular/liked to a party/group event |
| Getting a tattoo |  | Selection public transportation based on carbon emission |
|  |  | White lie |

##

##

##

##

##

##

##

##

##

##

##

##

**Table S2 | Determinants of norm updating strategies**


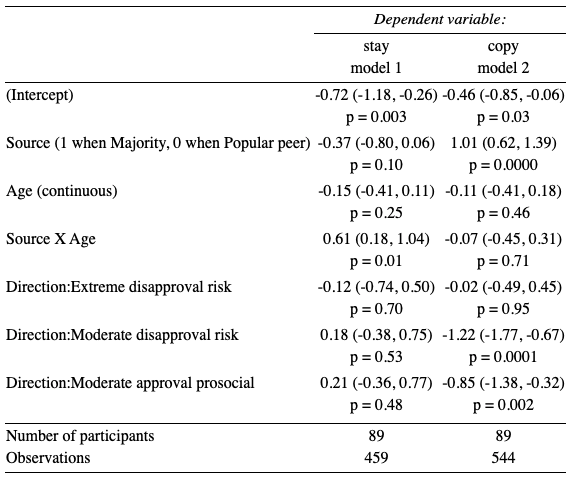


Logistic generalized mixed models were fitted to participants’ decisions to stay (model 1) and copy (model 2), using ‘participant’ as random intercept. Model 1 predicted participants’ probability to stay with their initial personal norm (coded as 1) or revising their personal norm towards the normative information (coded as 0). Model 2 predicted participants’ probability to copy the normative information (coded as 1) or do not copy it (coded as 0). Both models report unstandardized coefficients. The 95% confidence intervals (CI) are in parentheses and *P* values below the CI.

1. **Supplementary Methods**

Given that the original instructions of the experiment reported in our paper were in Dutch (the native language of all participants in our study), we provide English instructions. The original instructions can be made available from the corresponding author upon request. The experimental task was completed in two waves with a three-week interval. Each participant was assigned a random generated ‘participant ID’. In wave 1, risk-taking and prosocial items were randomized per screen and between participants. In wave 2, the blocks of the social sources were presented in counterbalanced order. Each block consisted of 6 trials. The task was performed on a tablet. We represent each screen with horizontal lines and on-screen buttons will be placed between square brackets.

**Wave 1**

**Welcome!**

Enter your participant number here:_______________

You will find this number on the summary of the information letter.

Make sure you fill it in correctly!

[Continue]

**Instructions 1 of 5**

In this game you rate **36 actions**.

We want to know what you think of each action.

We want to know if you disapprove or approve each action.

**For example**, It is not about whether you think that a certain action is bad for your health or the environment.

Instead, we want to know whether you would disapprove or approve that action.

[Continue]

**Instructions 2 of 5**

The choices you make will be used for a game in the second test session.

We will never show your choices to others with your name or other personal data.

Your classmates will therefore not know the choices you have made.

Your choices remain **anonymous**.

You make your choices by indicating what you think is bad (disapprove) or what you think is good (approve).

[Continue]

[Back]

**Instructions 3 of 5**

This game has **36 actions** divided by **6 screens**.

There are **6 actions** on each screen that you will assess.

You can make your choices on a screen that looks like this:


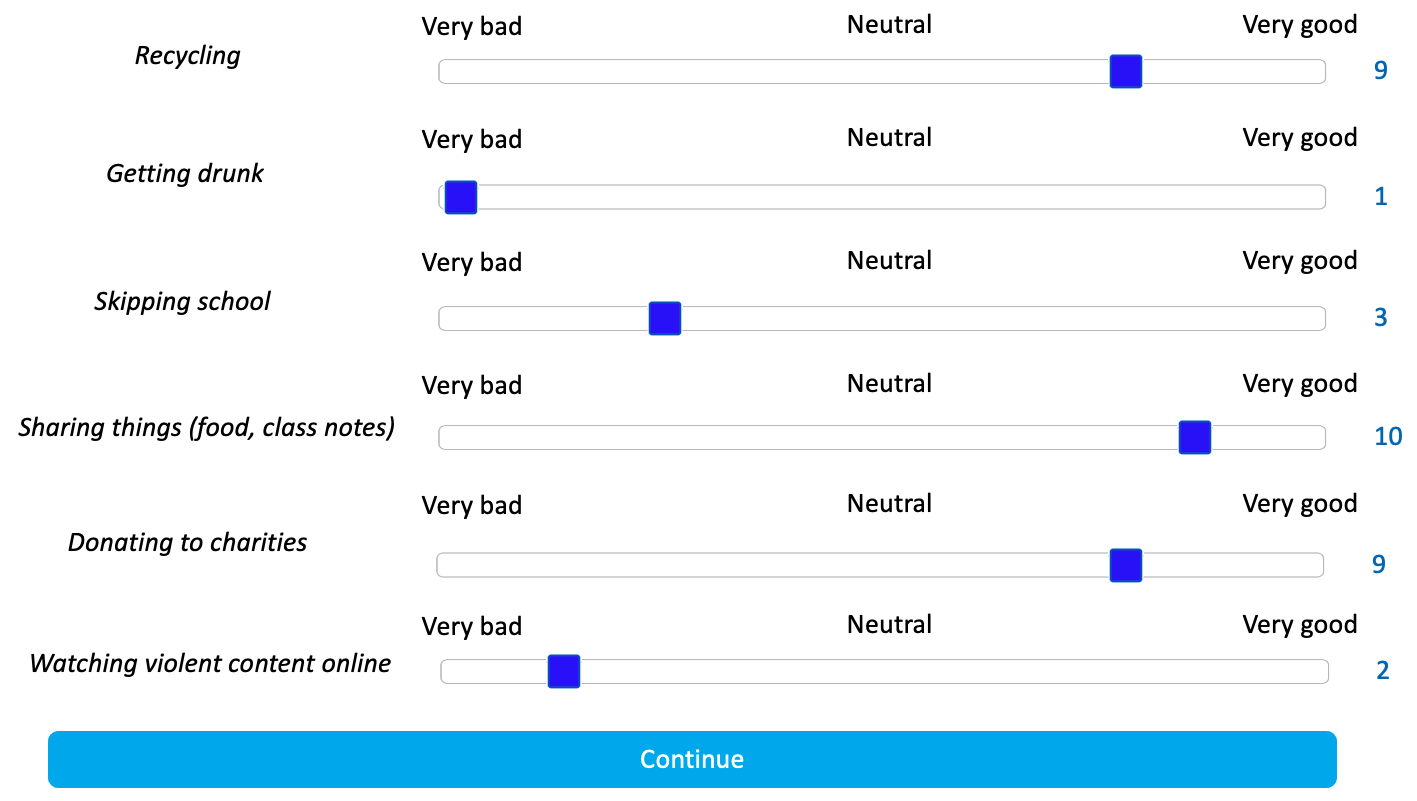


[Continue]

[Back]

**Instructions 4 of 5**

For each choice you see an action and a slider with which you can rate.

The slider is on a scale between 1 and 11.

**1** means '**Very bad**' and **11** means '**Very good**'.

[Continue]

[Back]

**Instructions 5 of 5**

Before starting this game, you can practice with the slider.

Here's an example of a slider:


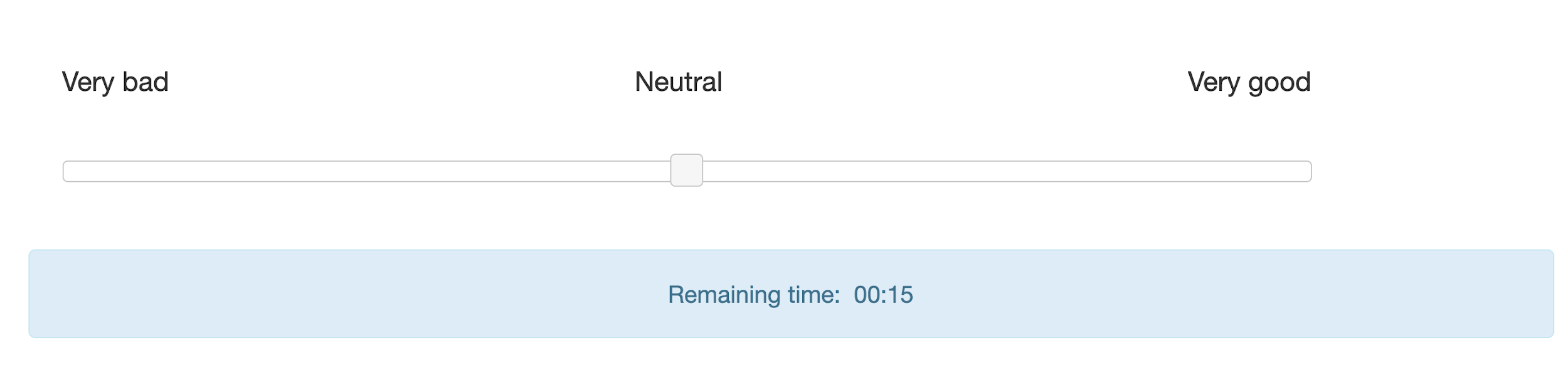


(participants had 15 seconds to get used to the slider and then the next screen would appear)

**Control questions**

We will now check if you understand the game.

Answer the following questions!

If you don't understand something, please call the experimenter.

1. How many choices do you have to make in total?

[32] [36] [28]

1. What does 1 mean in the slider?

[Very bad] [Good] [Bad]

1. What does 11 mean in the slider?

[Good] [Very good] [Bad]

[Continue]

[Back]

**Ready to start**

**Well done!**

You answered all control questions correctly!

Click on ‘**Start the game**’ below to start.

[Start the game]

**Screen 1** (example of a screen out 6)


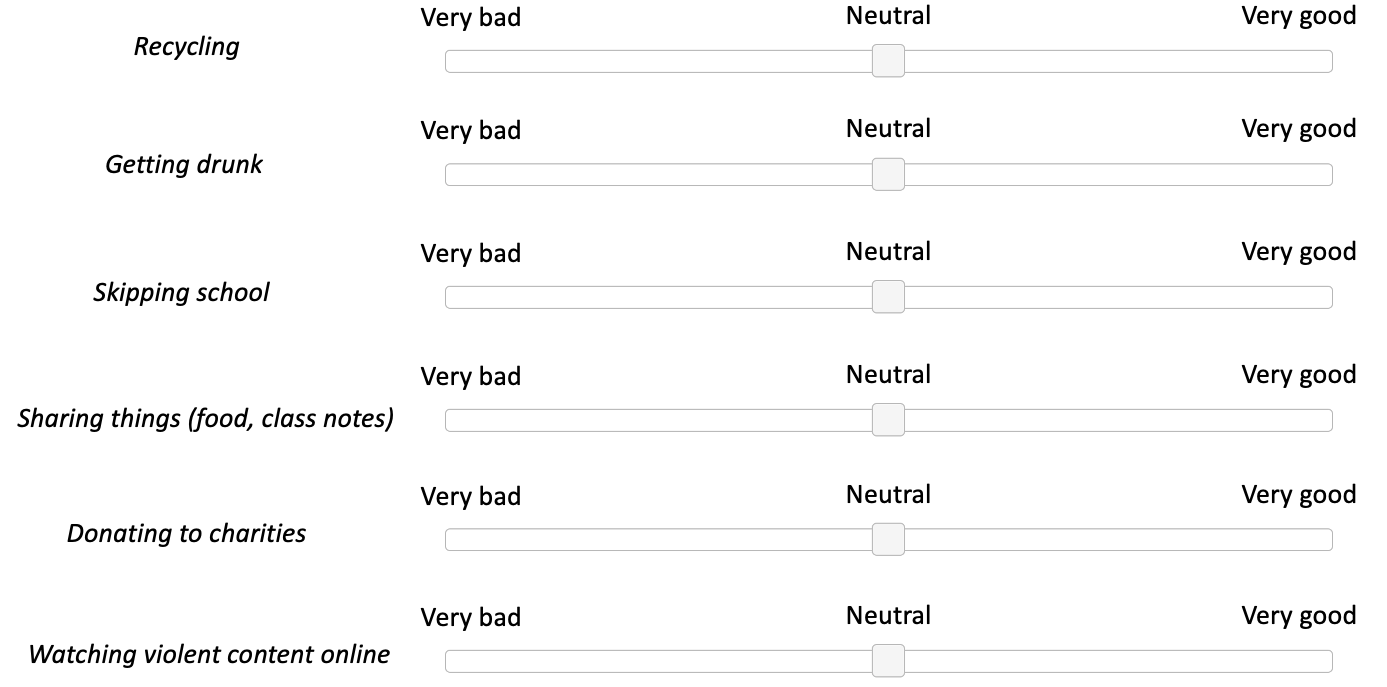


(a blue button would appear after participants had rated the items to go to the next screen)

**Session one of the game completed**

**End**

This is the end.

Thank you for your participation!

**Wave 2**

**Welcome!**

Enter your participant number here:

You will find this number on the summary of the information letter.

Make sure you fill it in correctly!

[Continue]

**Instructions 1 of 6**

In the first session of this game, you were shown 36 actions that you rated by using a slider.

We asked you to rate each action based on how you feel about it.

This is an example of what the screen looked like in the first session:

(here participants would see the same screen example as depicted above)

[Continue]

**Instructions 2 of 6**

In this session you will see **12 actions**.

We want to know what you think of each action.

We want to know if you disapprove or approve each action.

When you approve an action, you think that is appropriate for you or other people to do that action.

If you disapprove of an action, you feel that you or others should not do that, and that it is bad if they do.

**For example**, we want to know what you think and not whether something is good or bad for your health.

It may be that someone knows that smoking is bad for your health, but still feels it is appropriate to smoke alone or with others.

[Continue]

[Back]

**Instructions 3 of 6**

We have randomly selected 12 actions for you to rate again.

For 6 of these actions you will also see **the rating of one of the popular classmates**.

For the other 6 actions, you will see th**e rating that was most chosen in your classroom**.

[Continue]

[Back]

**Instructions 4 of 6**

Below is an explanation of '*a rating made by a popular classmate*'.

The popular classmate is a classmate who has been selected as popular more often than other classmates.

On a screen you will see the choice of a popular classmate for a certain action (green circle in the example).

Then you can drag the handle of the slider to what you think best suits the action you are seeing.

An example of a screen in which you see **the rating of a popular classmate** is:


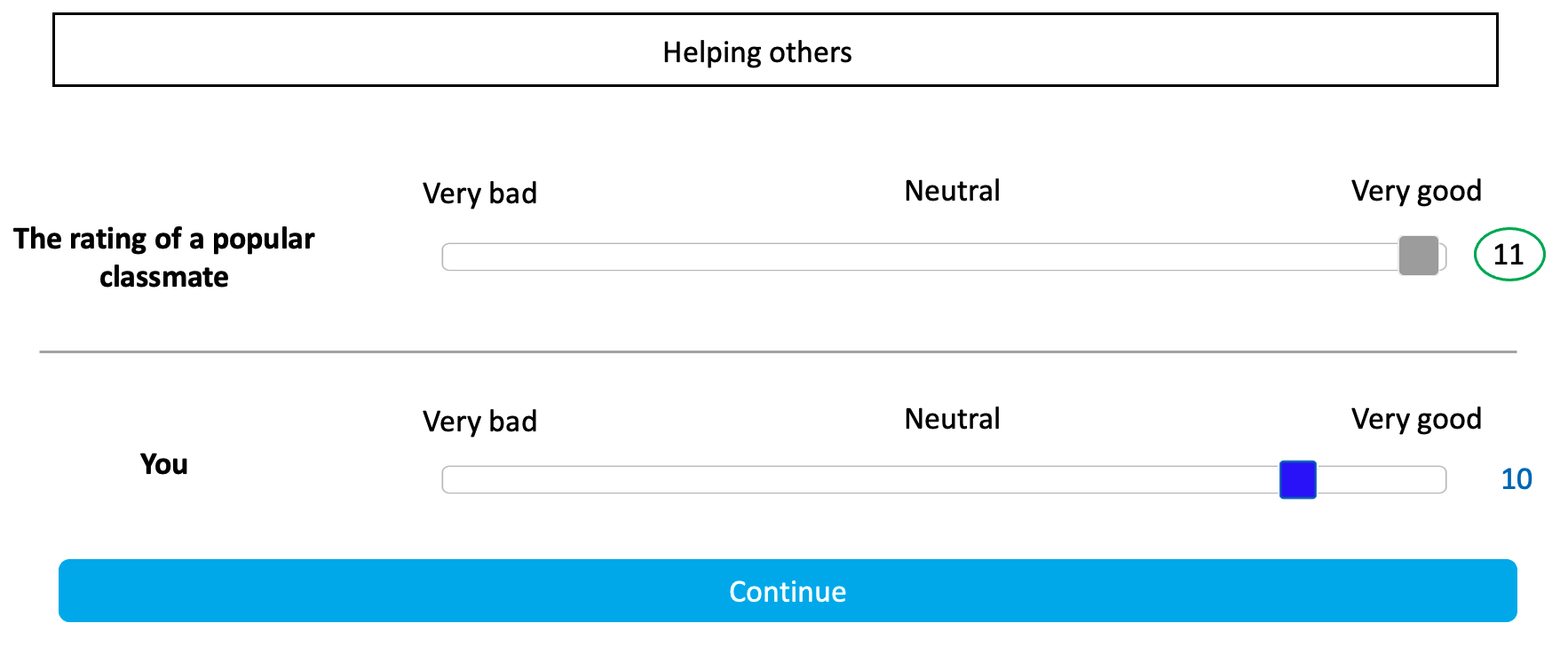


[Continue]

[Back]

**Instructions 5 of 6**

Below is an explanation of '*the most chosen rating in your classroom*'.

The choice most often made by your classmates is the one most selected for the action you see.

On a screen you will see the choice that was most often made by your classmates for a particular action (blue circle in the example).

Then you can drag the handle of the slider to what you think best suits the action you are seeing.

An example of a screen in which you see **the most chosen rating in your classroom** is:


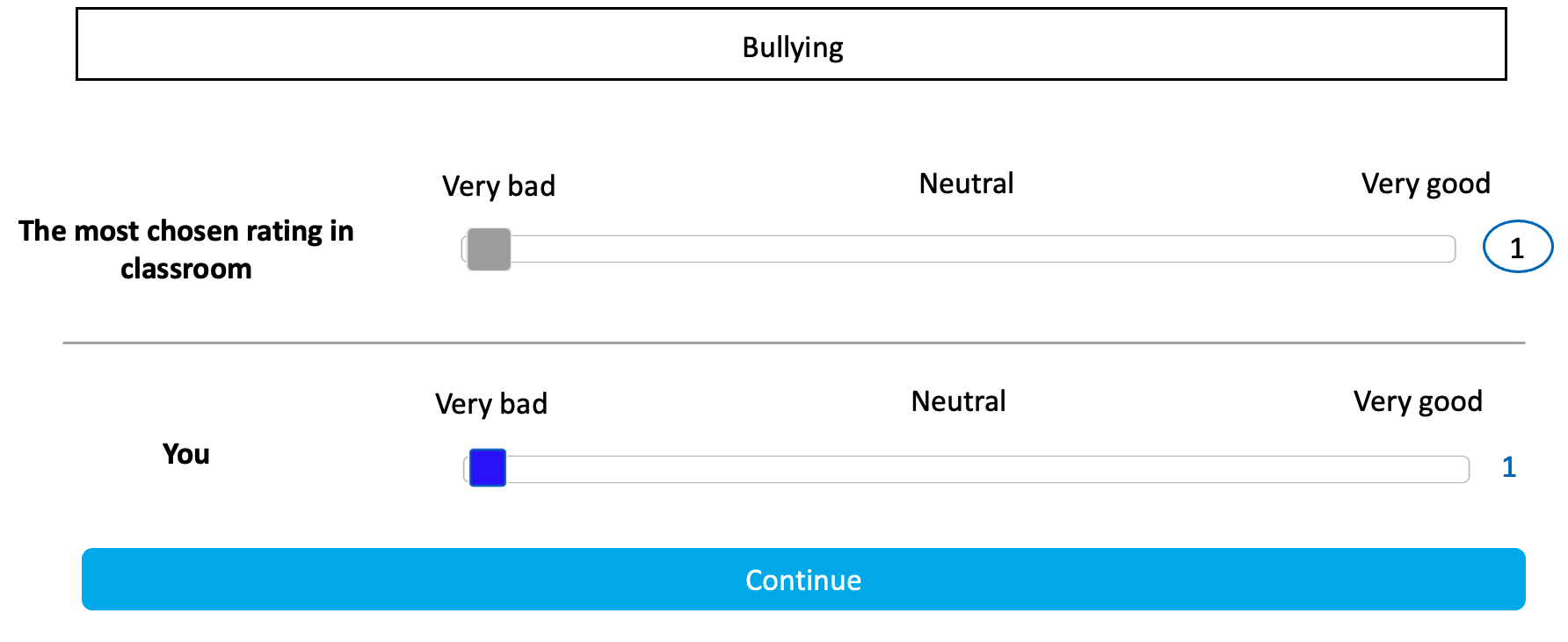


[Continue]

[Back]

**Instructions 6 of 6**

This game will contain **12 actions**.

6 actions followed by the choice of a popular classmate.

6 actions followed by a choice most selected by your classmates.

Under the actions and choices of others, you can use the slider to rate each action.

The slider goes from **1** to **11**.

**1** is ‘**Very Bad**’ and **11** is ‘**Very Good**’.

[Continue]

[Back]

**Control questions**

We will now check if you understand the game.

Answer the following questions!

If you don't understand something, please call the experimenter.

1. How many choices do you have to make in total?

[12] [36] [28]

1. What does 1 mean in the slider?

[Very bad] [Good] [Bad]

1. What does 11 mean in the slider?

[Good] [Very good] [Bad]

1. A popular peer is…

[a classmate who has been selected as popular more often than other classmates]

[a classmate who has been selected as popular less often than other classmates]

1. The most chosen rating in my classroom is..

[the least selected choice by my classmates]

[the most selected choice by my classmates]

[Continue]

[Back]

**Ready to start**

**Well done!**

You answered all control questions correctly!

Click on ‘**Start the game**’ below to start.

[Start the game]

**Block Popular peer** (one example of extreme peer disapproval of risk-taking)


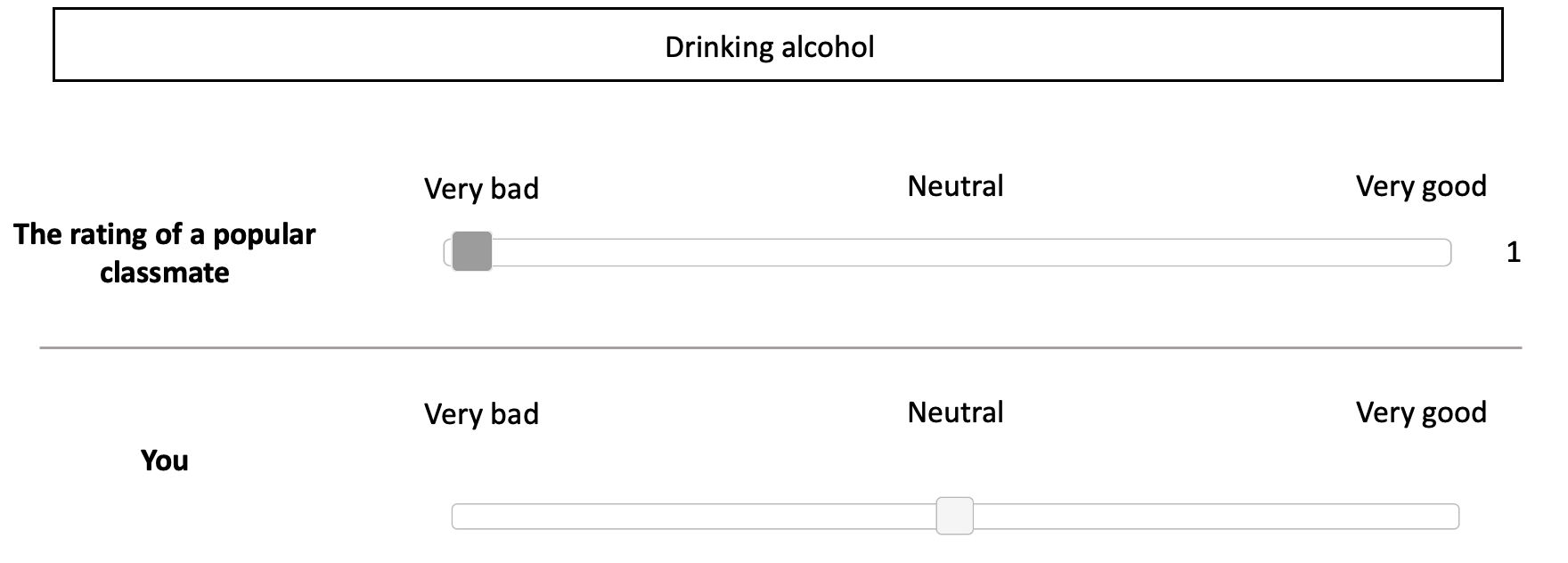


[a blue button would appear after participants had rated the item to go to the next screen]

**Block Popular peer** (one example of moderate peer approval of prosocial behaviour)


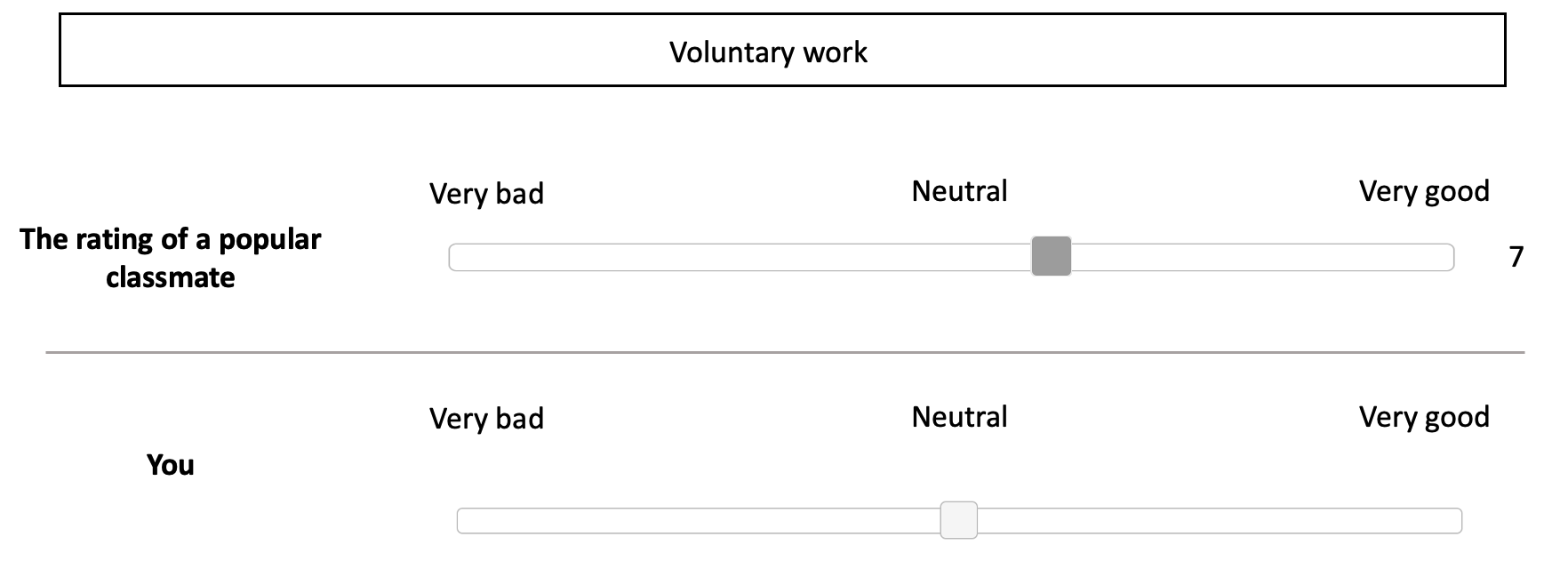


[a blue button would appear after participants had rated the item to go to the next screen; when the 6 trials of this block were completed, participants were presented with the other block]

**Block Majority** (one example of moderate peer disapproval of risk-taking)


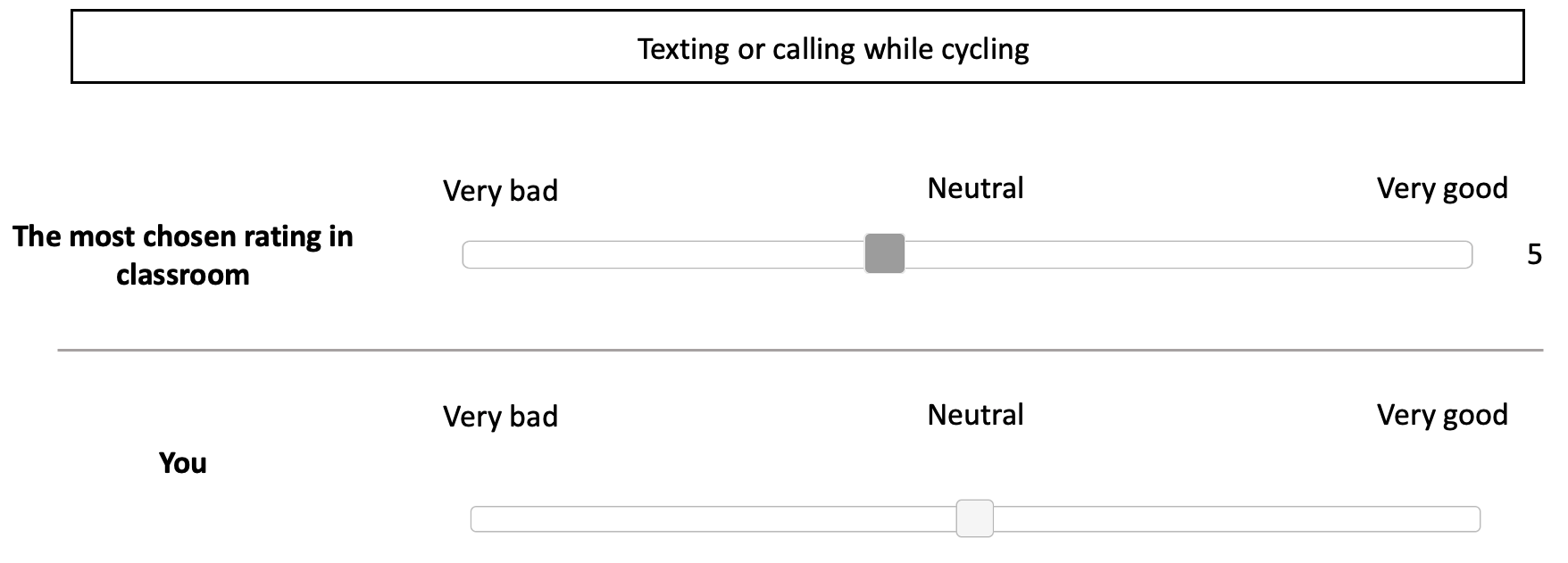


[a blue button would appear after participants had rated the item to go to the next screen]

**Block Majority** (one example of extreme peer approval of prosocial behaviour)


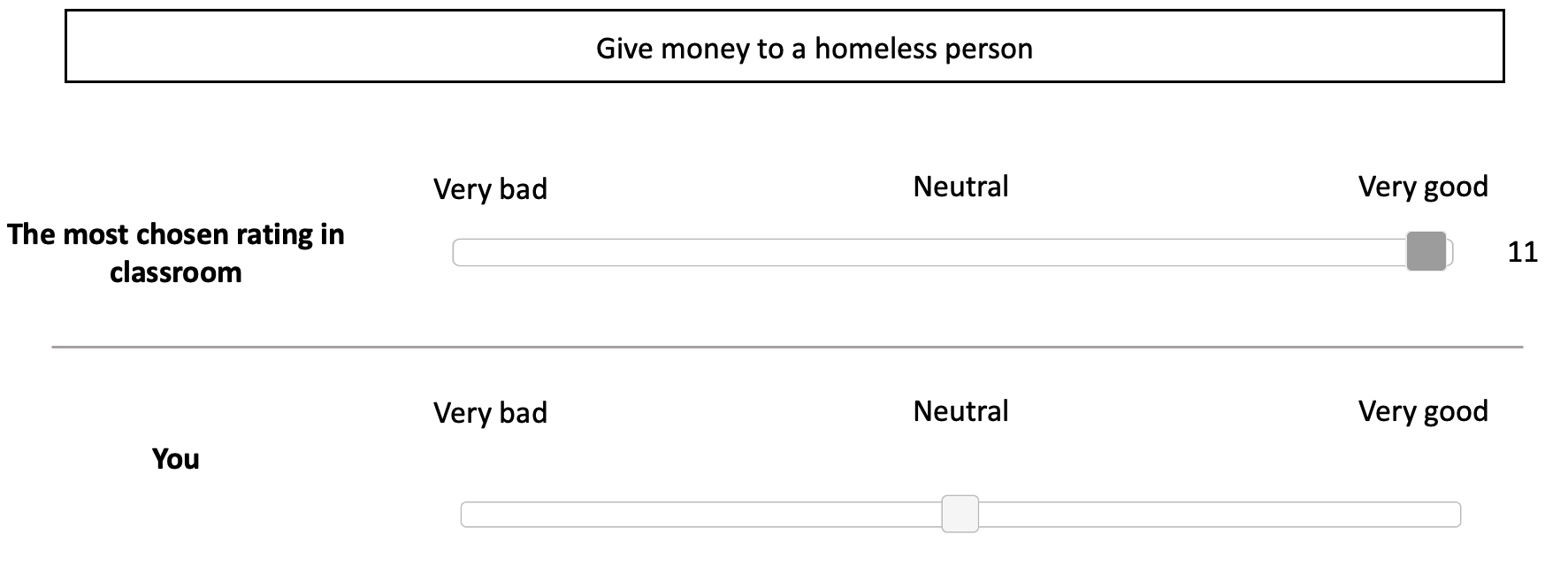


[after participants had finished the 6 trials a ‘continue’ button would appear to proceed to the last screen of the experiment]

**Game completed**

**End of this game**

This is the end of this game.

Thank you for your participation!
